# Juvenile idiopathic arthritis in Jordan: single center experience

**DOI:** 10.1186/s12969-021-00572-8

**Published:** 2021-06-12

**Authors:** Raed M. Alzyoud, Motasem O. Alsuweiti, Heba Q. Almaaitah, Bushra N. Aladaileh, Mohammad K. Alnoubani, Adel M. Alwahadneh

**Affiliations:** Pediatric Immunology, Allergy and Rheumatology Division, Queen Rania Children’s Hospital, Royal Medical Services, King Abdullah II St 226, P. O Box 11855, Amman, Jordan

**Keywords:** Juvenile idiopathic arthritis, Oligoarticular, ANA, Uveitis

## Abstract

**Background:**

Juvenile idiopathic arthritis (JIA) is a heterogeneous group of disorders, including all forms of arthritis, which develops in children who are less than 16 years old. This study aimed to evaluate the clinical and laboratory features of JIA in a single center in Jordan.

**Methods:**

A retrospective analysis of the electronic medical records of Pediatric patients diagnosed with JIA based on the International League of Associations for Rheumatology (ILAR) criteria during the period from 2015 to 2019 at the Pediatric Rheumatology Clinic in the Queen Rania Children’s Hospital. All patients were below the age of 14 years at the time of diagnosis and followed for at least 6 months. Collected data consisted of age, gender, age at initial presentation and diagnosis, JIA subtype, laboratory data, treatment options, and outcome.

**Results:**

A total of 210 patients were included in this cohort (94 males and 116 females) with the mean age at diagnosis and mean age at onset of 5.33 ± 3.40 years and 5.08 ± 3.40 years (range: 7 months – 14 years), respectively. Oligoarticular JIA was the commonest subtype (54.7%), followed by systemic arthritis (17.1%) and polyarticular arthritis (12.3%). ANA was positive in 70 patients (33.6%). Uveitis occurred in 30 (14.2%) patients.

**Conclusion:**

To the best of our knowledge, this study on this cohort is the first report on JIA in Jordan, in comparison with other regionally and internationally published reports. Oligoarticular JIA was found to be the most common subtype. For detailed knowledge on JIA characteristics and patterns, a population-based, rather than a single center study, should be conducted in Jordan.

## Background

Juvenile idiopathic arthritis (JIA) is an inflammatory disorder characterised by chronic arthritis and comprising all forms of arthritis that develop in children younger than 16 years of age. It lasts more than 6 weeks, with an unknown cause. JIA is still the most common disabling chronic rheumatic disease in children [1]. Less is known about the relationship between the genetic and environmental factors and the cause of the disease, responsible for disease heterogeneity [2].

Juvenile idiopathic arthritis has been classified by the International League of Associations for Rheumatology (ILAR) into the following seven subtypes: systemic, oligoarticular, rheumatoid factor (RF) positive and RF negative polyarticular, enthesitis-related arthritis (ERA), psoriatic, and ‘other’ JIA [3]. Published reports on JIA showed variable prevalence of JIA subtypes; oligoarticular JIA (27–56%), polyarticular RF negative JIA (11–28%), systemic JIA (4–17%), and ERA (3–11%) were the most common JIA subtypes [2]. Few reports from the Middle East and North Africa (MENA) described JIA among Arab children [4–10].

This study sought to describe JIA patterns in a single center and compare them with those from international and regional data. To the best of our knowledge, this was the first single center comprehensive study describing JIA in Jordan.

## Methods

A retrospective study was conducted in Queen Rania Children’s Hospital (QRCH), Amman, Jordan. Medical records of all patients who had been diagnosed with JIA from 2015 to 2019 were included; all patients were below 14 years of age at the time of diagnosis.

JIA diagnoses were made and classified according to ILAR classification criteria [3] by a Pediatric rheumatologist based on available information recorded during the study period at each clinic visit. Only patients with at least 6 months follow-up duration were included to have more details about JIA characteristics. The study was approved by the local ethical committee of the Royal Medical Services.

Data on gender, age at disease onset, patient age at the time of diagnosis, joint involvement at presentation, systemic manifestations and JIA subtype, and treatment options were gathered. Laboratory data included data on total white blood cell (WBC) count (leukocytosis defined as WBC > 11 × 10^3^/uL), haematocrit (Ht) level (anaemia defined as Ht < 31%), platelet (PLT) count (thrombocytosis defined as PLT > 450 × 10^9^/L), elevated C-reactive protein (CRP) concentration > 3 mg/L, erythrocyte sedimentation rate (ESR) > 20 mm/hr., ANA positivity, and rheumatoid factor (RF).

ANA was determined by indirect immunofluorescence using Hep-2 cells; titre > 1/80 was considered positive. RF was studied on nephelometry and considered positive when titre was ≥15 units/mL. The RF-positive disease was determined by the attainment of at least two positive results, 3 months apart, in the first 6 months of observation.

All patients were screened for uveitis, frequency of visits based on uveitis risk, using a slit-lamp examination at a dedicated uveitis clinic.

American College of Rheumatology (ACR) Recommendations for the Treatment of Juvenile Idiopathic Arthritis 2013 [11] were followed in the treatment of our patients. Remission was determined based on the preliminary criteria published by Carol Wallace [12].

For the analysis of continuous outcome data, we used the analysis of variance (ANOVA) test with post-hoc analysis using the Tukey correction. Pearson’s chi-square test, Fisher’s exact test, and odds ratios (OR) were used to assess the distribution of categorical variables. Results were considered significant for a *p*-value of less than 0.05. Statistical analysis was performed using the Statistical Program for Social Science (SPSS) version 18.0 (Armonk, NY).

## Results

A total of 210 patients with JIA (94 (45%) males and 116 (55%) females; male-to-female ratio, 1:1.2) were included in this study. The mean age at diagnosis was 5.33 ± 3.4 years (range: 7 months to 14 years).

Twenty-four (11.4%) patients had a positive family history of rheumatoid arthritis; 37 (17.6%) patients had a consanguineous parent. Demographic data are shown in Table 1.

**Table 1:** Demographics of 210 patients

	Range	Male (*n* = 94)	Female (*n* = 116)	Total (*n* = 210)
Age at disease onset (mean)	7 mo - 14 yr	5.42 ± 3.3 yr	4.90 ± 3.4 yr	5.08 ± 3.4 yr
Age at diagnosis (mean)	8 mo - 14 yr	5.77 ± 3.4 yr	5.0 ± 3.3 yr	5.33 ± 3.4 yr
Follow-up duration (mean)	6–203 mo	–	–	45.3 ± 37.9 mo
Consanguinity	–	24 (25%)	13 (11%)	37 (17.6%)
Family history of RA	–	8 (8.5%)	16 (14%)	24 (11.4%)

*Yr* years, *mo* months, *wk* week, *RA* rheumatoid arthritis

JIA subtype data in our cohort are shown in Table 2. With 115 (54.7%) patients, oligoarthritis was the most prevalent subtype, followed by systemic arthritis (36 (17.1%) patients), polyarthritis (26 (12.3%) patients), psoriatic arthritis (18 (8.5%) patients), and enthesitis-related arthritis (15 (7.1%) patients). Patients with oligoarticular JIA were sub-classified into persistent oligoarticular JIA (96 (84%) patients) and extended oligoarticular JIA (19 (16%) patients).

**Table 2:** Distribution of JIA subtypes by gender and age of onset

JIA subtype	n (%)	Gender		Age of onset (yr)	
		Male	Female	Range	Mean
Oligoarthritis	115 (54.7%)	39 (34%)	76 (66%)	10 mo- 14 yr	5.0 ± 3.2 yr
R.F (−) polyarthritis	18 (8.5%)	6 (33%)	12 (66%)	4 yr. −12 yr	8.0 ± 3.2 yr
R.F (+) polyarthritis	8 (3.8%)	2 (25%)	6 (75%)	4 yr. −12 yr	8.0 ± 3.2 yr
Systemic arthritis	36 (17.1%)	20 (56%)	16 (44%)	9 mo- 13 yr	3.8 ± 2.7 yr
Enthesitis-related arthritis	15 (7.1%)	9 (60%)	6 (40%)	6 yr- 12 yr	10 ± 1.77 yr
Psoriatic arthritis	18 (8.5%)	10 (56%)	8 (44%)	7 mo- 11 yr	5.5 ± 3.9 yr

*JIA* Juvenile Idiopathic Arthritis, *RF* Rheumatoid factor, *mo* months, *yr* years

**Fig. 1:**
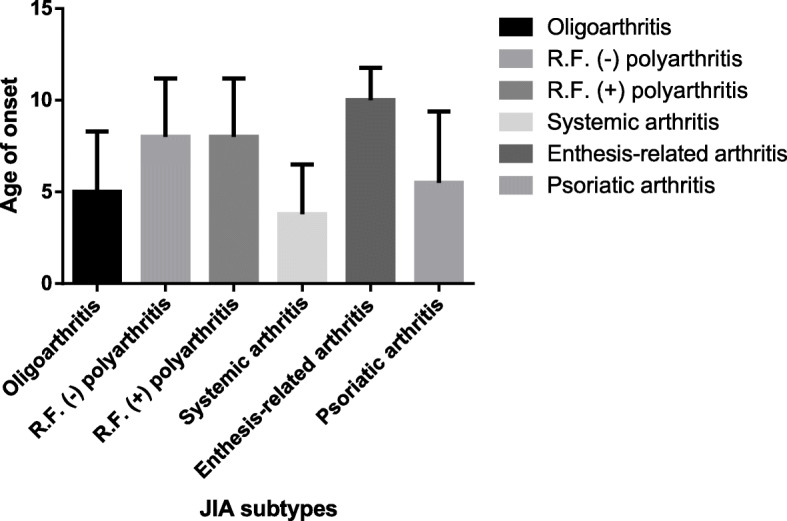
Mean age in different JIA subgroups

For oligoarticular JIA, female predominance was noticed, as females had a higher frequency (76 (66%) patients) with a male-to-female ration of 1:1.94. The mean age at disease onset of oligoarticular JIA was 5.0 ± 3.4 (range: 10 months to 14 years) years; it differed significantly between JIA subtypes (*P*-value < 0.01), with oligoarthritis and systemic arthritis having an earlier onset (Fig. 1).

Systemic arthritis was observed to be the second most frequent subtype. Thirty -six (17.1%) patients had systemic arthritis; of them, 20 (56%) and 16 (44%) patients were males and females, respectively.

Data on the clinical presentations of the patients are listed in Table 3. The most common joint involvement at presentation was the knee, which occurred in all systemic arthritis, polyarticular JIA, and 80% of oligoarticular JIA. The ankle joint followed with frequencies of 21, 30, and 50% in oligoarticular, polyarticular, and systemic JIA, respectively. Wrist arthritis was observed in 12, 11.5, and 55.5% of patients with oligoarticular, polyarticular, and systemic JIA, respectively. Whereas elbow arthritis was found at presentation in 21, 15, and 28% of patients with oligoarticular, polyarticular, and systemic JIA, respectively.

**Table 3:** Articular and extraarticular clinical manifestations at presentation

	Oligoarticular JIA n (%)	Polyarticular JIA n (%)	Systemic JIA n (%)	*P*-value
TMJ	4 (3.4%)	0 (0%)	0 (0%)	n/a
Cervical	0 (0%)	0 (0%)	0 (0%)	n/a
Shoulder	0 (0%)	0 (0%)	0 (0%)	n/a
Elbow	24 (21%)	4 (15%)	10 (28%)	0.48
Wrist	14 (12%)	3 (11.5%)	20 (55.5%)	< 0.001
MCP	0 (0%)	4 (15%)	1 (2.7%)	n/a
PIP	1 (0.8%)	3 (11.5%)	2 (5.5%)	n/a
Hip	2 (1.6%)	1 (4%)	0 (0%)	n/a
Knee	92 (80%)	26 (100%)	36 (100%)	< 0.001
Ankle	24 (21%)	8 (30%)	18 (50%)	< 0.01
Fever	0 (0%)	0 (0%)	36 (100%)	n/a
Rash	0 (0%)	0 (0%)	24 (66.6%)	n/a
Lymphadenopathy	0 (0%)	0 (0%)	2 (5.5%)	n/a
Hepatosplenomegaly	0 (0%)	0 (0%)	0 (0%)	n/a
Serositis	0 (0%)	0 (0%)	2 (5.5%)	n/a
Uveitis	25 (21.7%)	3 (11.5%)	1 (2.7%)	0.02
MAS	0 (0%)	0 (0%)	2 (5.5%)	n/a

*P*-values are based on the Chi-square test performed to assess the difference in the distribution of each manifestation between the subgroups. n/a: not applicable; where there were not enough cases in each subgroup to perform the analysis. *p* is significant for less than 0.05

*TMJ* temporomandibular joint, *MCP* metacarpophalangeal joint, *PIP* proximal interphalangeal joint, *MAS* macrophage activation syndrome, *n/a* not applicable

In systemic arthritis, fever at presentation was reported in all patients; skin rash occurred in 24 (66.6%) patients, while lymphadenopathy and serositis were reported in 2 (5.5%) patients for each. Macrophage activating syndrome (MAS) occurred in only 2 (5.5%) patients.

Uveitis was reported in 30 (14.2%) patients; most of them had oligoarticular JIA (25 (21.7%) patients). Uveitis was associated with ANA positivity in 16 (14%) patients. Additionally, it was reported in 1 (2.7%) and 3 (11.5%) patients with systemic and polyarticular arthritis, respectively.

We calculated the distribution of articular and extra-articular clinical manifestations (see Table 3) for oligoarticular, polyarticular, and systemic JIA. We only compared the manifestations where we had at least one recorded case for each subgroup, meaning: elbow, wrist, knee, ankle, and uveitis. Pearson’s chi -square test revealed a significant difference in the distribution of symptoms among the groups (*P* < 0.05).

Odds ratios (ORs) were computed for each subgroup pair (Oligo-JIA/Poly-JIA; Oligo-JIA/Systemic JIA; Poly- JIA/Systemic JIA). We applied Fisher’s exact test for independence, and reported the *p*-value, OR, and OR 95% confidence interval (CI), as presented in Table 4.

**Table 4:** Odds ratios between the subgroups for extraarticular manifestations

	Oligo JIA/Poly JIA			Oligo JIA/Systemic JIA			Poly JIA/Systemic JIA		
	***p***-value	OR	95% CI	***p***-value	OR	95% CI	***p***-value	OR	95% CI
Elbow	0.78	1.45	0.45–4.61	0.37	0.685	0.29–1.61	0.35	0.472	0.12–1.72
Wrist	1	1.063	0.28–4.006	< 0.0001	0.11	0.046–0.262	0.0005	0.104	0.026–0.411
Knee	0.007	0.07	0.004–1.26	0.002	0.053	0.003–0.91	1	0.726	0.013–37.81
Ankle	0.3	0.59	0.23–1.52	0.001	0.26	0.119–0.583	0.19	0.44	0.15–1.28
Uveitis	0.28	2.130	0.59–7.67	0.009	9.722	1.26–74.55	0.3	4.565	0.44–46.64

*P*-values represent the results of Fisher’s exact test applied for each manifestation between two groups. A *p* < 0.05 was considered significant and show that in our dataset, the extraarticular manifestation and the subgroups are dependent

*CI* confidence interval, *JIA* juvenile idiopathic arthritis, *OR* odds ratio

Elbow arthritis was independent of the subgroups (*P* > 0.05). Wrist arthritis was dependent on the subgroups for Oligo-JIA/Systemic JIA and Poly-JIA/Systemic JIA (*P* < 0.0001). Patients with systemic JIA had higher odds of developing wrist arthritis than patients with oligo and poly-JIA. Knee arthritis was dependent on the Oligo-JIA/Poly-JIA and Oligo-JIA/Systemic JIA subgroups (*P* < 0.05). Patients with oligo-JIA had lower odds of developing these symptoms than the other subgroups. For ankle and uveitis manifestations, a dependence on subgroups was observed only in the Oligo-JIA/Systemic JIA category (*P* < 0.001), with ankle involvement being more common for systemic JIA and uveitis in oligo-JIA.

The most common laboratory findings at diagnosis in our cohort were elevated acute phase reactants (ESR, 34%; CRP, 32%), followed by leukocytosis (16%). In systemic arthritis, anaemia (19%), leukocytosis (38%), thrombocytosis (36%), and elevated acute phase reactants (CRP, 75%; ESR, 66%) were observed. Positive ANA was present in 70 (33.3%) patients, and mostly occurred in patients with the oligoarticular subtype (61 (53%) patients), with female predominance (47 (77%) female patients). Additionally, it was reported positive in three (8.3%) patients with systemic arthritis. Details of the main laboratory investigations are listed in Table 5.

**Table 5:** Main laboratory investigations at diagnosis

	Total n (%) mean (range)	Systemic arthritis n (%) mean (range)	Oligoarticular n (%) mean (range)	Polyarticular n (%) mean (range)	Psoriatic arthritis n (%) mean (range)	ERA n (%) mean (range)
Anemia Hct < 31%	20 (10) 34.6 (23.8–42.5)	7 (19) 34 (23.8–41)	9 (8) 34.6 (23.8–41)	3 (11) 34.7 (32.3–36.3)	1 (5) 36.9 (34.9–41)	0 (0) 36.2 (34.9–42.5)
WBC > 11 X 109/L	35 (16) 10.9 (4.9–39.4)	14 (38) 13.3 (5.7–39.4)	20 (17) 10 (4.9–18.2)	0 (0) 7.5 (7.0–8.3)	0 (0) 9.2 (7.2–11)	1 (6) 9.2 (5.7–12.6)
PLT > 450 X 109/L	32 (15) 421.4 (127–1076)	13 (36) 460 (127–1076)	16 (14) 409 (201–895)	0 (0) 297 (236–356)	1 (5) 387 (285–524)	2 (12) 415 (301–598)
ESR > 20 mm/hour	72 (34) 51 (5–140)	24 (66) 51 (9–140)	39 (34) 50.9 (5–123)	3 (11) 99 (79–128)	1 (5) 35 (22–60)	5 (33) 61.4 (15–100)
CRP > 3 mg/L	69 (32) 37 (0–284)	27 (75) 67 (0–284)	32 (27) 24 (0–283)	3 (11) 75 (48–89.7)	3 (16) 6 (0–6)	4 (26) 19.8 (0–45.8)
ANA	70 (33.3)	3 (8.3)	61 (53)	4 (15)	0	2 (13.3)
RF	8 (3.8)	0	0	8 (30)	0	0

*Hct* hematocrit, *WBC* white blood cells, *PLT* platelets, *ESR* erythrocyte sedimentation rate, *CRP* c-reactive protein, *ANA* antinuclear antibody, *RF* rheumatoid factor, *ERA* enthesitis related arthritis

Pharmacological treatments for patients during the study period are listed in Table 6. Non-steroidal anti-inflammatory drugs (NSAIDs) were used in 174 (82.8%) patients. Steroids were administered to majority of the patients (191 (91%) patients)—oral (160 (76.1%) patients), intravenous (IV) (33 (15.7%) patients), and intra-articular (136 (64.7%) patients). Disease-modifying anti-rheumatic drugs (DMARDs) were used in 198 (94.2%) patients, and methotrexate was the most common DMARD used (171 (81.4%) patients). Biological treatment was used in half of the patients (105 (50%) patients). Infliximab was the most common biologic used in 30 (14.2%) patients.

**Table 6:** Pharmacological Treatment used in all JIA subtypes

Subtype	Oligoarticular	Polyarticular	Systemic arthritis	Psoriatic arthritis	Enthesitis Related arthritis	Total (%)
Drug						
NSAIDs	102	9	34	15	14	174 (82.8%)
steroid	110	17	36	15	8	191 (91%)
oral	84	17	36	15	8	160 (76.1)
IA	96	8	26	4	2	136 (64.7%)
IV	6	8	17	2	0	33 (15.7%)
MTX	87	26	32	18	8	171 (81.4%)
HCQ	4	0	0	0	0	4 (1.9%)
MMF	1	0	0	0	0	1 (0.5%)
leflunomide	4	0	0	0	2	6 (2.8%)
Cyclosporine	0	0	5	2	0	7 (3.3%)
Sulfasalazine	2	0	0	0	0	2 (0.95%)
Infliximab	12	5	6	3	4	30 (14.2%)
Etanercept	14	0	2	7	2	25 (12%)
Adalimumab	12	0	2	1	0	15 (7.1%)
Tocilizumab	2	0	15	0	0	17 (8%)
Anakinra	0	0	1	0	0	1 (0.5%)
Rituximab	0	2	5	1	0	8 (3.8%)
Golimumab	2	2	0	0	0	4 (1.9%)
Secukinumab	0	0	0	3	2	5 (2.3%)

*NSAIDs* non-steroidal anti-inflammatory drugs, *IA* intra-articular, IV: intravenous, *MTX* methotrexate, *HCQ* hydroxychloroquine, *MMF* Mycophenolate mofetil

The mean follow-up period in our cohort was 45.3 ± 37.9 months (range: six–203 months). According to preliminary criteria published by Wallace [12], 145 patients achieved inactive disease; of these, 117 (56%) and 28 (13%) patients were in remission on and off medication, respectively. Systemic arthritis has the highest percentage of remission on medication (61%), whereas the highest remission off medication was reported in polyarticular JIA. Remission status of patients in the study in different JIA subgroups is demonstrated in Table 7.

**Table 7:** Remission status of patients in the cohort study

	number	Active disease	Remission on medication	Remission off medication
Oligoarticular	115	34 (30%)	65 (56%)	16 (14%)
Polyarticular	26	8 (31%)	12 (46%)	6 (23%)
Systemic arthritis	36	8 (22.2%)	22 (61%)	6 (16.6%)
Psoriatic arthritis	18	8 (44.4%)	10 (55.5%)	0
Enthesitis-related arthritis	15	7 (46.6%)	8 (53.3%)	0
Total	210	65 (31%)	117 (56%)	28 (13%)

Active disease, remission on medication and remission off medication means the status at 1 year after diagnosis

## Discussion

This retrospective study was conducted in the Pediatric Rheumatology Division at Queen Rania Children’s Hospital, Amman, Jordan, which is the only center in the country dedicated to Pediatric rheumatology disorders. To the best of our knowledge, this was the first single center comprehensive study describing JIA in Jordan.

According to published international reports, oligoarticular JIA is the most common JIA subtype, as reported in Spain (51%), Sweden (44.7%), and Turkey (41%) [13–15]. Similarly, oligoarticular JIA was the most common subtype in our cohort with a frequency of 115 (57.7%) patients. With regard to published data from some countries in the Middle East and North Africa (MENA), such as Lebanon (31%), Iraq (48%), Saudi Arabia (40.5%), and Egypt (41.3%), oligoarticular JIA was the most common subtype [4–7]. Whereas other reports from MENA found polyarticular JIA to be the most common subtype in Oman (46.7%) [8] and Tunisia (66%) [9].

We compared our data in this study with those from countries in MENA and other regions, as shown in Table 8.

**Table 8:** The pattern of JIA in different countries

Country	Current Study *N* = 210	Saudia Arabia [10] *N* = 82	Saudia Arabia [6] *N* = 74	Lebanon [4] *N* = 66	Iraq [5] *N* = 52	Eqypt [7] *N* = 196	Oman [8] *N* = 107	Tunisia [9] *N* = 54	Turkey [15] *N* = 634	Sweden [14] *N* = 251	Spain [13] *N* = 145
Subtype											
Oligoarthritis	54.70%	28.04%	40.5%	31%	48.08%	41.30%	31.80%	15.10%	41%	44.70%	51%
Polyarthritis	12.38%	29.26%	32.40%	24%	36.54%	34.70%	46.70%	66%	23.50%	20.70%	12.40%
Systemic arthritis	17.14%	36.50%	8.10%	23%	9.62%	24%	17.80%	7.60%	14.50%	2.80%	6.90%
Enthesitis related arthritis	7.14%	1.21%	1.40%	17%	5.77%	0%	2.80%	9.40%	18.90%	8.80%	12.40%
psoriatic arthritis	8.57%	4.87%	1.40%	0%	0%	0%	0.90%	1.90%	2.10%	6.80%	6.20%
Undifferentiated arthritis	0%	0%	16.20%	5%	0%	0%	0%	0%	0%	16.30%	11.10%

The current study reported that the mean age at disease onset was 5.08 ± 3.40 years (range: 7 months to 14 years), which was much lower than that reported by Abou El-Soud et al. [16] (mean: 10.5 ± 3.60 years; range: 4–15 years), but similar to that published by Bahabri et al. [17] (mean: 6 years). It was also lower than the mean age of European origin patients as published by Saurenmann et al. [18] (mean: 6.5 years; range: 6.1–6.8 years). This observation might be explained by the younger age range of the Pediatric population of our cohort, as patients older than 14 years are seen by adult rheumatology. The current study showed female predominance (116 female patients; male-to-female ratio: 1:1.2); a higher ratio (1:1.7) was reported by Solau-Gervais et al [19]

According to Table 5, elevated acute phase reactant levels (CRP and ESR) were the most common findings, unlike the report by Al-Hemairi et al. [10], where anaemia was the most common finding at diagnosis (59.7%). Compared to our study, anaemia was observed in only 10%. Whereas in systemic arthritis, anaemia, leukocytosis, and thrombocytosis had much higher percentages than those observed in our study.

ANA was positive in 33.6% of cases, although others, such as Khuffash et al. [20] and Ozdogan et al. [21] recorded fewer numbers (12 and 5%, respectively). This observation reflected on the higher incidence of uveitis. ANA positivity was highest among patients with oligoarticular JIA (61 (53%) patients), which is similar to findings (50%) by Al Wahadneh et al [22]

Oligoarthritis is an overwhelming disease of the lower limbs, with the knee joint being the most part affected, followed by the ankle joint [23]. Similar to data recorded globally, the pattern of joint involvement in oligoarticular JIA in our study had a lower limb predominance, with the knee and ankle joints involved in 80 and 21% of all cases, respectively. Similar joint involvement was found in polyarticular JIA, with the knee and ankle joints involved in 100 and 30% of all cases, respectively.

Extra-articular manifestations in systemic-onset JIA were fever (reported in all patients), followed by skin rash (66.6%). The pattern of joint involvement showed upper and lowered joint involvement, with knee and ankle arthritis reported in 100 and 50%, respectively, whereas elbow and wrist involvement were 28 and 55.5% of all cases, respectively. Our study reported a higher prevalence of both articular and extra-articular manifestations in systemic arthritis than the study from Egypt [24].

Uveitis was reported in 30 (14.2%) patients, which was comparable to results (11.6%) published by Angeles-Han et al [25] Oligoarticular JIA was the most common subtype associated with uveitis (25 (21.7%) patients). ANA positivity was found in 16 (64%) patients. According to a study from Saudi Arabia, uveitis was observed in 8.1% of patients with oligoarticular JIA [26]. Another large population-based study in Germany on patients with JIA reported that uveitis occurred in 12% of all JIA subtypes (extended oligoarticular (25%) and persistent Oligoarticular (16%)) [27]. However, whether if this complication is due to the high prevalence of oligoarticular JIA is unclear.

MAS is a life-threatening complication of systemic JIA [28]. In this study, it was observed in only two (0.9%) patients, whereas Çakan et al. [29] reported a higher incidence (33.9%) of this serious complication. Çakan et al. [29] explained that this high rate of MAS was because the study was conducted in a referral center for Pediatric rheumatology and the high percentage of Mediterranean fever (MEFV) gene mutation carriers, which may increase the possibility of developing more auto-inflammatory disorders than in other healthy populations. JIA treatment aims to reduce pain, gain joint function, preserve muscle strength, and avoid systemic complications [30]. Although no consensus on JIA treatment has been reached, many guidelines have been established by different rheumatology societies or colleges. In our Pediatric rheumatology division, we follow the American College of Rheumatology (ACR) recommendations for the JIA treatment [11]. Based on ACR recommendations, we followed the plan to start treatment with NSAIDs, and in case of inadequate response, conventional DMARDs were started. In case of failure, no response, or intolerance, we switched to another DMARD or added a biological agent.

NSAIDs have traditionally been the mainstay treatment for all kinds of JIA during the first 4-6 weeks of initial treatment, either alone or with combination with intra-articular steroid injection [31]. NSAIDs were used in 174 (82.8%) patients—oligoarticular JIA (103 (89.2%) patients) and systemic JIA (35 (96.7%) patients—at diagnosis or during their disease course. A higher percentage of 99% of patients with oligoarticular JIA in central Italy received NSAIDs [32].

Systemic steroids are used in the treatment of JIA at diagnosis or during disease flare [33]. In our cohort, systemic steroids were used in 91% of patients. A short course of low dose systemic steroids was used as bridging therapy in 127 patients with oligoarticular and polyarticular JIA at the time of diagnosis or during disease flare and in all patients with systemic arthritis to control systemic manifestation.

Methotrexate (MTX) is the cornerstone treatment in oligoarticular, polyarticular, and systemic JIA with articular inflammation predominance [34]. In our cohort, MTX was used in 81.4% of the cases— 76.7, 100, and 90% of cases in oligoarticular, polyarticular, and systemic JIA, whereas MTX was used in 66% of cases in the Omani study [8]. Other DMARDs were used in cases of MTX toxicity or intolerance; leflunomide, sulfasalazine, myfortic (MMF), and hydroxychloroquine were administered in 6 (2.8%), 2 (0.95%), 1, and 4 (1.9%) cases, respectively.

Biological treatment has been found to be safe and effective in severe JIA or refractory cases to synthetic DMARDs [35]. In our cohort, biological agents were used in 105 (50%) patients. These agents include anti -TNF (tumour necrosis factor), which is a cytokine that plays a role in the pathogenesis of JIA and found in higher levels in the synovial fluid. Tocilizumab, a monoclonal antibody directed against IL-6 receptor, increased serum levels in systemic arthritis. Anakinra is a human recombinant IL-1 receptor antagonist, plays a role in the pathogenesis of JIA, and is a preferred treatment option for systemic arthritis. Rituximab is a human monoclonal antibody directed against CD20 lymphocytes, which results in increased B-cell apoptosis and decreased levels of mature B cells expressing CD20 [36]. In our cohort, anti -TNF drugs were used in 74 (35.2%) patients. Infliximab (30 (14.2%) patients) was the most common anti-TNF used, followed by etanercept (25 (12%) patients). Whereas adalimumab and golimumab were used in 15 (7.1%) and 4 (1.9%) patients, respectively. In comparison, biological DMARDs were used in 28.4% of patients from Saudi Arabia, with adalimumab being the most common biological treatment [6]. This difference could be due to the larger size of our cohort and a more severe disease at the time of diagnosis in our cohort. Thirty-eight (18%) patients in our cohort used more than one biological agent, and this group of patients reflects the more severe course in our cohort. Most of them switched to another biological agent due to the inefficiency of the previous agents.

Tocilizumab was used in 17 (8%) patients—13 patients with systemic arthritis. Anakinra (IL-1 antagonist) was used in one (0.5%) patient with systemic arthritis (Anakinra is not yet registered in Jordan, and it was offered to the patient from outside the country). Rituximab was used in 8 (3.8%) patients—five patients were with systemic arthritis refractory to DMARDs, steroids, and other biological treatments. Alexeeva et al. [37] showed that rituximab may be effective in severe systemic arthritis resistant to immunosuppressive treatment, glucocorticoid therapy, and other biological treatments.

In our cohort, 31% of all patients showed active disease during the last follow-up. Remission on medication was observed in 56%, and off medication in 13%; whereas Shen et al. [38] reported patients with active disease, remission on medication, and off medication during the last follow-up (40, 14.9, and 45.1%, respectively). A similar outcome was reported by Chhabra et al. [39], where inactive disease was reported in 73% (remission on medication (25%) and remission off medication (47%). The low percentage of remission off medication can be explained by difficulty in achieving remission off medication in both psoriatic and enthesitis-related arthritis.

Our study limitations included the design (being a retrospective study), setting (single center rather than population-based, which may give more details about JIA characteristics), and limited severity spectrum (that it did not include ‘mild cases’, which are not referred to our hospital or misdiagnosed). In addition, we did not include patients with JIA who were older than 14 years at disease onset due to governmental policy of Pediatric age cut-off, contrary to ILAR’s definition.

## Conclusion

To the best of our knowledge, this was the first study in Jordan describing the clinical and laboratory characteristics of JIA, considering that our hospital is the country’s leading tertiary clinic for Pediatric rheumatology. These results show the pattern of JIA in a single center. Unlike in countries in the Arabian Gulf and North Africa, oligoarticular JIA was the commonest subtype, as recorded in some Middle East and European countries.

## Data Availability

The datasets used and/or analyzed during the current study are available from the corresponding author on reasonable request.

## Abbreviations

JIA: Juvenile idiopathic arthritis
ILAR: International League of Associations for Rheumatology
ANA: Antinuclear antibody
RF: Rheumatoid factor
NSAIDs: Non-steroidal anti-inflammatory drugs
DMARD: Disease-modifying anti-rheumatic drugs
MMF: Myfortic
MTX: Methotrexate
